# An Exploratory Study of the Safety and Efficacy of a *Trigonella foenum-graecum* Seed Extract in Early Glucose Dysregulation: A Double-Blind Randomized Placebo-Controlled Trial

**DOI:** 10.3390/pharmaceutics14112453

**Published:** 2022-11-14

**Authors:** Emily Pickering, Elizabeth Steels, Amanda Rao, Kathryn J. Steadman

**Affiliations:** 1 School of Pharmacy, University of Queensland, Brisbane, QLD 4102, Australia; 2 Evidence Sciences Pty, Ltd., Brisbane, QLD 4005, Australia; 3 School of Human Movement and Nutrition Sciences, University of Queensland, Brisbane, QLD 4102, Australia

**Keywords:** *Trigonella foenum-graecum*, fenugreek, prediabetes, type 2 diabetes, fasting blood glucose, post-prandial glucose, insulin

## Abstract

Background: This was an exploratory study to assess the safety and efficacy of a specialized *Trigonella foenum-graceum* L. seed extract for supporting healthy blood glucose metabolism in a pre-diabetic cohort. Methods: Fifty-four participants were randomised to receive 500 mg/day of *T. foenum-graecum* seed extract or matching placebo daily for 12 weeks. Fasting blood glucose (FBG), post-prandial glucose (PPBG), HbA1c, fasting insulin (FI), post-prandial insulin (PPI) and C-peptide were assessed at baseline, week 6 and week 12. Lipid levels, liver enzymes and C-reactive protein (CRP), along with safety markers and tolerability were also assessed at baseline and week 12. Results: By week 12 there was a significant difference in FBG (*p* < 0.001), PPBG (*p* = 0.007) and triglycerides (*p* = 0.030) between treatment groups, with no changes in HbA1c (*p* = 0.41), FI (*p* = 0.12), PPI (*p* = 0.50) or C-peptide (*p* = 0.80). There was no difference in total cholesterol (*p* = 0.99), high-density lipoprotein (*p* = 0.35), low density lipoprotein (*p* = 0.60) or CRP (*p* = 0.79). There was no change in safety markers and the treatment was well tolerated. Conclusions: The results of the study indicated that *T. foenum-graecum* seed extract may influence blood glucose metabolism and larger studies are warranted to evaluate efficacy and potential mechanisms of action.

## 1. Introduction

Type 2 diabetes mellitus (T2DM) is a growing epidemic across the world and currently accounts for over 90% of all world-wide cases of diabetes, equating to approximately 483 million people in 2021 and predicted to rise to 705 million by 2045 [1]. The preceding condition, termed prediabetes, is a major risk factor for developing T2DM and is characterized as either or both impaired fasting blood glucose (IFG) and/or impaired blood glucose tolerance (IGT). Over 319 million people (6.2% of the world population) are recorded to have IFG, with predictions to rise to 440 million (6.9%) by 2045, while the impact of IGT is higher again, with 541 million (10.6%) currently affected and predicted to rise to 730 million (11.4%) by the year 2045 [1]. In addition, the prediabetic condition poses its own potential health risks as IGT is associated with metabolic syndrome as well as being a strong predictor of atherosclerotic cardiovascular disease, potentially increasing the development cardiovascular disease by approximately 15% [2].

Insulin resistance and uncontrolled calorie consumption are recognized as key contributors to the conversion of prediabetes to T2DM [1]. Treatments for prediabetes include dietary and lifestyle changes as first-line strategies. Interventions that reduce insulin resistance and the need for insulin secretion by pancreatic beta-cells, such as glucose-lowering medications (i.e., metformin) may also reduce the likelihood of prediabetes progressing to T2DM [3]. Other interventions such as functional foods and herbal medicines are also an ongoing area of research interest for managing early glucose dysregulation.

Herbal remedies, such as *Trigonella foenum-graceum*, *Gymnema sylvestre,* and *Momordica charantia* have been used as preventative measures and as treatments for managing glucose dysregulation [4]. The seeds of some plants, such as *Linum usitatissimum* L. (flax), *Salvia hispanica* (chia) and *T. foenum-graceum* (fenugreek) have been used as ingredients to help lower glycemic loads of foods, thus reducing the glycemic impact of carbohydrates and simple sugars [5,6]. *T. foenum-graecum,* from the family Fabaceae (syn Leguminosae), is a culinary herb, the seeds of which have traditionally been used in the management of blood glucose levels [7]. There is support from clinical studies of *T. foenum-graecum* seeds in treating early and established T2DM as a food ingredient [6,8,9], raw powdered seeds added to water [10,11,12] and seed extracts [13,14,15,16]. In a recent clinical trial, 10 g/day of a *T. foenum-graecum* powder was found to have a protective effect against progression from prediabetes to T2DM when used long term [17]. Despite the large body of previous studies, including a meta-analysis [18], showing *T. foenum-graceum* seeds can reduce blood glucose levels in those type T2DM, less research exists regarding *T. foenum-graceum* seed extracts as a treatment for prediabetes. It is important to distinguish the effects of *T. foenum-graceum* seed extracts between those with T2DM and prediabetes as efficacy with the lower blood glucose levels featured in prediabetes may differ from that of T2DM. The safety of *T. foeneum-graceum* seed has previously been assessed in those with T2DM as both raw seed [19] and seed extracts [20] and is generally considered to not cause any adverse or intolerable effects. This is yet to be established in those with prediabetes. 

The aim of this study is to assess the efficacy and safety of a specialised *T. foenum-graecum* seed extract in supporting healthy glucose metabolism in adults diagnosed with prediabetes (IFG and/or IGT) as a stand-alone treatment, over a 12-week period.

## 2. Materials and Methods

### 2.1. Trial Design

This clinical trial was a double-blind, placebo-controlled randomized study of participants who were diagnosed with pre-diabetes, and not currently prescribed antidiabetic medications, to assess the effectiveness of *T. foenum-graecum* extract on reducing fasting blood glucose (FBG) and post-prandial blood glucose (PPBG) and associated metabolic parameters over 12 weeks. The study was conducted in Brisbane, Australia between March 2018 and March 2020 and was carried out according to the principles expressed in the Declaration of Helsinki. It was approved by the Bellberry Ethics Committee No: 2017-08-601 and registered with the Australian New Zealand Clinical Trials Registry (ANZCTR) No: ACTRN12618000031268.

### 2.2. Investigational Products

The investigational product was a dark green film-coated tablet containing 250 mg of *T. foenum-graecum L.* seed, hydro-alcoholic extract (marketed by Gencor Pacific Ltd., Lantau Island, Hong Kong, under the brand name Trigogen™), standardized to no less than 20% trigonelline and no less than 20% amino acids which was identified by high performance thin layer chromatography (HPTLC) by Indus Biotech Pty Ltd. The placebo product was an identical dark green film-coated tablet containing maltodextrin. The products were provided by Gencor Pacific Ltd., Unit 3, 1/F, Office Building Block 2, 96 Siena Avenue, Discovery Bay North, Lantau Island, N.T., Hong Kong. Participants took either the investigational product or the placebo tablets, twice a day (one in the morning and one in the evening) for 12 weeks. 

### 2.3. Study Outcome Measures

Primary outcome: The primary outcome was evaluation of fasting blood glucose (FBG) as part of the standard 2 h oral glucose tolerance test (2hOGTT) at baseline, 6 weeks and 12 weeks. FBG was used to assess IFG.

Secondary outcomes: The secondary outcomes included post-prandial blood glucose (PPBG) using the standard 2hOGTT at baseline, 6 weeks and 12 weeks. PPBG was used to assess IGT. Fasting insulin (FI), and post-prandial insulin (PPI) were also assessed at baseline, 6 weeks and 12 weeks. The other secondary outcomes were measured at baseline and 12 weeks: glycated haemoglobin (HbA1c), C-peptide, C-reactive protein (CRP) and lipids (total cholesterol, triglycerides (TG), low density lipoprotein (LDL), high density lipoprotein (HDL)) and the safety markers; full blood count (FBC) and a comprehensive metabolic panel (including electrolytes, liver, and kidney function) at baseline and at 12 weeks. The Depression Anxiety Stress Scale (DASS-21) was used to assess stress, anxiety and depression levels of participants at baseline and 12 weeks [21]. Product tolerability was evaluated at clinic interviews by investigators at all clinic appointments. An adverse event (AE) was defined as any untoward medical occurrence and that does not necessarily have a causal relationship with this treatment, and safety testing procedures followed the NHMRC Safety Monitoring Guidelines and Reporting in Clinical Trials involving Therapeutic Goods, 2016 [22].

### 2.4. Inclusion, Exclusion, and Screening

Inclusion criteria: Participants were included if they had a FBG > 5.5 mmol/L or a PPBG of > 7.8 mmol/L and agreed to have a 2hOGTT on three (3) occasions; at baseline, 6 weeks and 12 weeks. The cut-off levels for FBG and PPBG matched the classification system used in Australia for diabetes screening and diagnosis at the time of the study [23].

Exclusion criteria: Those with a normal glucose profile or a previous diagnosis of T2DM, were outside healthy weight reference range of 18 to 35 BMI or were taking anti-obesity medications or oral blood glucose-lowering medications such as sulfonylureas, biguanides, alpha-glucosidase inhibitors, thiazolidinedione or taking any dietary supplements or herbal medicines specifically for management of blood glucose levels were excluded. Other exclusions included recent episodes of symptomatic coronary arterial disease, stroke, any cardiovascular events including infarction, treatment for cancers within last 5 years, those taking anticoagulants, those with known past reactions to *T. foenum-graecum* seeds, cucumber or maltodextrin, those with an admission of alcohol abuse or the use of illicit drugs, and pregnancy or breastfeeding or not having had a normal menstrual cycle. Potential participants were also excluded if they were considering commencing new lifestyle interventions, including changing diet or physical activity for the purpose of managing body weight or blood glucose levels, or if they had completed participation in any other clinical trial during past month.

Screening: The initial screening process (screening 1), conducted online and through telephone screening, identified potential participants that met all inclusion and exclusion criteria and were: (1) at risk of T2DM on the Australian Type 2 Diabetes Risk Assessment Tool (AUSDRISK) questionnaire, score > 6, or (2) were those that had been medically diagnosed as pre-type 2 diabetic (pre-diabetic) by their medical practitioner. These potential participants underwent further screening (screening 2) and were invited to undertake an in-clinic FBG test (taken at 8 am, after fasting the previous night from 10 pm), using handheld Accu-Chek glucose meters. They were deemed eligible to participate in the study if they had a FBG > 5.5 mmol/L at this time. Assessment of the inclusion criteria of PPBG of > 7.8 mmol/L was only possible with the baseline 2hOGTT, and in a prediabetic state FBG levels are still variable, so enrolled/consented participants that were found in the assessments made at baseline to have either a normal FBG, or PPBG > 7.8 mmol/L were subsequently excluded and referred to their medical practitioner.

### 2.5. Randomisation, Blinding and Study Procedures

The randomisation of the products (1:1 ratio, in blocks of 30) was performed independently of the investigators using computer generated random allocation software. The products were blinded independently of the investigators. The trial products were delivered to the investigators in trial product containers that were identical in function and appearance, with the only difference being the trial number on the labels of the containers. The investigators allocated the next available numbered container in the sequence as each participant enrolled in the study, ensuring that all participants had an equal chance of receiving the investigational product or the placebo product. The investigators and participants were therefore blinded for the duration of the study.

Study procedures: At the baseline visit, study procedures were explained, and participants provided informed written consent. The participant provided health information including health history, current medications/supplements, and had body weight, height and blood pressure measured. Participants were provided with their allocated trial product and were instructed to maintain the same diet and level of physical activity for the duration of the study. Prior to commencing the product, participants were asked to attend an independent pathology clinic for the baseline pathology tests, which were 2hOGTT (for IFG, PPBG, FI and PPI), HbA1c, C-peptide, lipid studies, CRP, FBC and a comprehensive metabolic panel were completed by an independent laboratory and results provided to investigators via an online portal. For the 2hOGTT, participants attended the pathology clinic at 8 am in a fasted state (no food since 10 pm the previous night). A blood draw was taken at time 0 (baseline), followed by ingestion of a flavoured 70 g glucose drink (within 5 min) and then subsequent blood draws at 1 and 2 h post-prandial to measure both blood glucose and insulin levels. At week 6 the participants were assessed for compliance and for adverse events and completed a 2hOGTT. At week 12 (final interview), participants returned all containers and any unused products and had the third (final) pathology test which was a repeat of the baseline pathology tests. Compliance was monitored throughout the study by the trial investigators and participants were considered compliant if they had taken over 80% of the product.

### 2.6. Sample Size and Data Analysis

Pre-study, it was determined that a sample size of 58 participants would be required to detect a 0.37 mmol/L change in FBG between active treatment group and placebo group over 12 weeks, assuming a 0.4 mmol/L change score, at 80% power and a type I error of 5%. A total of 57 participants were enrolled, as recruitment was suspended in March 2020 due to the COVID-19 pandemic, resulting in 48 participants being included in the analysis. Post-study, a 0.37 mmol/L difference in FBG was observed with the smaller sample size. The effect size was calculated from the difference between the two means to provide a quantitative measure of the magnitude of the experimental effect and to be reflective of clinical significance. Analysis of the effect size for the primary outcome (FBG) was calculated using Cohen’s D, and found to be d = 1.108, indicating a large effect size with > 79% of control group measuring higher than the mean of the experimental group, thus making the sample size sufficient for this study.

All data is expressed as mean ± standard deviation unless otherwise specified. A modified intent to treat (ITT) approach was used for data analysis of the primary outcome, where all participants who completed midpoint pathology were included in the analysis. The ITT single missing data points for pathology data was managed using the simple imputation method, with the data from the last observation carried forward. Normality between groups was assessed using the Kolmogorov–Smirnov and Sharpiro-Wilk tests. The glucose pathology parameters FBG, FI, PPBG and PPI, and DASS-21 were assessed as change scores from baseline to week 12 and analyzed using 2-sided student *t*-tests. All other pathology tests were assessed by difference between groups at baseline and 12 weeks using 2-sided *t*-tests. Cohen’s d test was used to determine effect size and correlations were performed using Pearson’s coefficient using the Statistical Packages for the Social Sciences (SPSS) software version 21 (statistical significance set at *p* < 0.05). 

## 3. Results

### 3.1. Demographics

**Figure 1:**
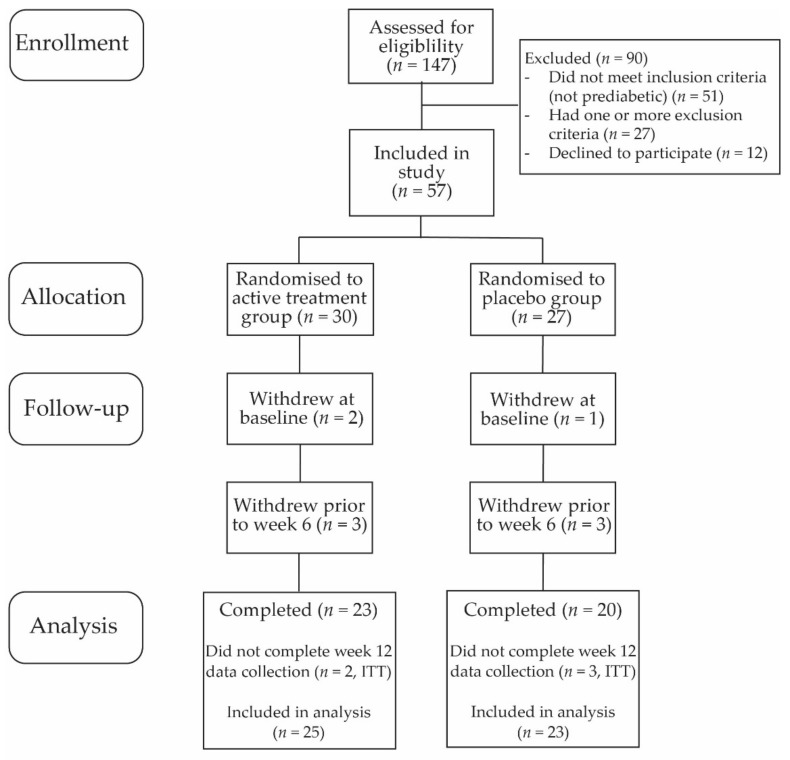
CONSORT participant flow chart.

The study initially enrolled 57 participants (Figure 1, Table 1). Of the 30 participants allocated to active treatment group, 25 were included in analysis, with the five withdrawals due to being inducted but not completing baseline (*n* = 2), not pre-diabetic on baseline pathology assessment (*n* = 1), withdrew prior to week 6 (*n* = 1) and an adverse event (*n* = 1). There were 27 participants allocated to placebo group, with 23 participants included in analysis, and 4 withdrawals due to not being pre-diabetic on baseline pathology assessment (*n* = 1) or withdrew prior to 6 weeks (*n* = 3). Participants included these previously identified as pre-diabetic by their medical practitioner (active treatment group, 60%; placebo group, 35%), with the remainder being those who were found to be prediabetic upon screening. 

### 3.2. Effect of Treatment on Glucose Metabolism Markers

**Figure 2:**
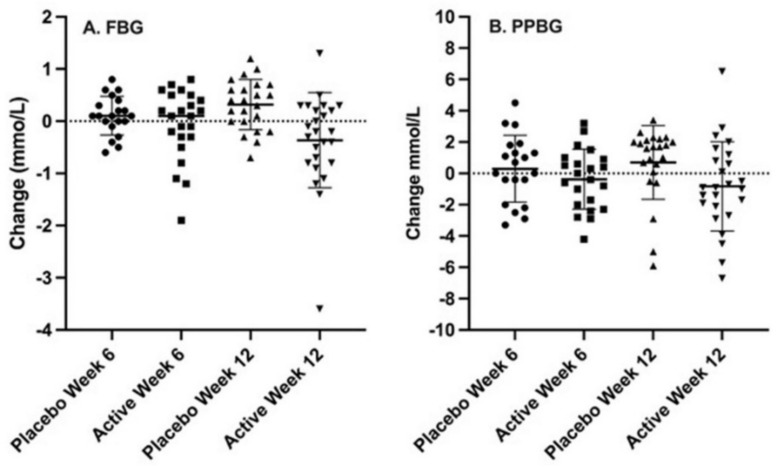
Change from baseline in fasting blood glucose levels ((**A**) FBG) and 2 h post prandial blood glucose levels ((**B**) PPBG) for Active treatment group and Placebo group at week 6 and week 12.

Fasting blood glucose (FBG): The baseline FBG levels appeared to be slightly higher for the active treatment group (7 mmol/L) than the placebo group (6 mmol/L) but this was borderline non-significant (*p* = 0.052) (Table 2). There was no difference in FBG between groups at 6 weeks (*p* = 0.20), however, by 12 weeks a statistically significant difference was observed (*p* < 0.001) (Figure 2A, Table 2). 

Post-prandial blood glucose (PPBG): Baseline PPBG levels, assessing blood glucose in the fed state, were similar for both groups at one and two hours post-prandial (*p* = 0.31, and *p* = 0.21, respectively) (Table 2). While the 1 h PPBG levels in the active treatment group showed a trend of reduction at 6 weeks and further reduction at 12 weeks, reflecting a total reduction of 0.73 mmol/L, the placebo group remained stable (Table 2). The 2 h PPBG in the active treatment group steadily reduced through the study and was significantly different from baseline. In contrast, in the placebo group, the 2 h PPBG levels increased steadily over the study period (Figure 2B), with the groups being significantly different by 12 weeks (*p* = 0.007).

HbA1c levels: The baseline HbA1c levels correlated with baseline FBG levels (r = 0.944). HbA1c levels were similar for both groups at baseline (*p* = 0.15) and were not statistically different at 12 weeks (*p* = 0.41) (Table 3). 

C-peptide: C-peptide levels were similar between the active treatment and placebo groups at baseline (*p* = 0.075) and also stayed similar at week 12 (*p* = 0.79) (Table 3). 

Insulin levels: The fasting insulin (FI) levels varied widely, as expected in a prediabetic state. The mean FI appeared to be slightly higher in the active treatment group though this was not significant (*p* = 0.20) and may reflect the corresponding slightly higher FBG also observed at baseline (Table 2). The FI levels in the active treatment group had a greater reduction by 12 weeks in contrast to the placebo group although this was not significantly different (*p* = 0.14). Both the 1 h and 2 h postprandial insulin (PPI) levels were similar between groups at baseline, 6 weeks and 12 weeks (Table 2).

### 3.3. Effect of Treatment on Blood Lipids, C-Reactive Protein and DASS-21

The total cholesterol levels, HDL and LDL levels were similar in both groups at baseline and week 12 (Table 3). The triglyceride levels were similar at baseline (*p* = 0.23), with a significant difference observed between groups after 12 weeks (*p* = 0.030) due to an increase in levels for the placebo group and no change in those taking the active treatment. C-reactive protein, used as marker of inflammation, was not significantly different between groups at baseline or 12 weeks (Table 3). The assessment of mood (DASS-21) indicated that both groups reported similar normal to low levels of depression (active, 2.28 vs. placebo, 1.52, *p* = 0.20), anxiety (0.76 vs. 1.30, *p* = 0.31) and stress (2.68 vs. 1.83, *p* = 0.15) at baseline, with no significant changes at week 6 (depression: -0.44 vs. −0.44, *p* = 0.99; anxiety: −0.28 vs. −0.48, *p* = 0.51; stress: −0.12 vs. −0.09, *p* = 0.95) or week 12 (depression: −0.80 vs. −0.52, *p* = 0.56; anxiety: −0.24 vs. −0.70, *p* = 0.27; stress: 0.04 vs. −0.52, *p* = 0.28).

### 3.4. Safety, Adverse Events and Tolerability

Safety was assessed by the FBC and a comprehensive metabolic panel (including electrolytes, liver and kidney function). These markers were in healthy reference range for both groups at baseline and week 12 (Table 4). There were no reported changes in diet or physical activity during the study. There was a single report of feeling “light-headed”, possibly hypoglycemia, resulting in withdrawal from the study, with no other adverse events reported.

## 4. Discussion

The results of this study demonstrated, in a group of pre-diabetic adults, that 500 mg per day of this hydro-alcoholic *T. foenum-graceum* seed extract was associated with a significant positive effect on fasting and post-prandial blood glucose levels against worsening FBG levels in the placebo group. In addition, those taking the *T. foenum-graceum* extract maintained triglyceride levels while the placebo group had an increase in triglyceride levels over the course of the 12-week study, indicating that the condition was progressing in those with untreated prediabetes. 

The results of this study support a previous long-term study in pre-diabetics using 10 g of powdered *T. foenum-graceum* seeds taken daily for three years, showing a protective effect in the prevention of T2DM in those with pre-diabetes [17]. The beneficial reduction of FBG levels by *T. foenum-graceum* seed preparations has also been witnessed in adults with T2DM. In a study that added 10 g of *T. foenum-graceum* seeds soaked in hot water alongside anti-diabetic medication in 60 patients with T2DM, there was a greater reduction in fasting blood glucose levels than with the anti-diabetic medications alone [12]. *T. foenum-graceum* (8 g/day of powdered dried seeds for 8 weeks) was also advantageous as an add-on to exercise in men with T2DM with a reduction in FBG and 2 h PPBG at a level greater than exercise or *T. foenum-graceum* alone [24]. The mechanism of action in these whole seed studies is likely linked to the fiber content, as has been found with other seeds such as flaxseed [25] chia [5]. 

Extracts of *T. foenum-graceum* seeds differ from the abovementioned studies, in that they do not contain fiber. A hydro-alcoholic *T. foenum-graceum* seed extract at a dose of 1 g per day for 12 weeks significantly reduced FBG levels compared to standard care (dietary control and exercise) alongside placebo in 25 patients with newly diagnosed T2DM [13]. A saponin-rich *T. foenum-graceum* seed extract at 6.3 g per day as an add-on to oral sulphonylurea medication in 69 poorly controlled T2DM patients was reported to maintain blood glucose levels, and significantly decrease FBG and PPBG levels [14]. A more recent RCT study of 154 people with T2DM assessing 1 g of a patented furostanolic saponin-enriched *T. foenum-graceum* seed extract as an add-on medication to oral anti-diabetic prescription also reported a significant decrease in FBG and 2 h PPBG glucose after 12 weeks [20]. 

In this study, there was no reduction in HbA1c levels over the 12 weeks. There are conflicting results for the effect of *T. foenum-graceum* seeds on HbA1c levels in T2DM conducted over a similar time of 12 weeks, with positive results recorded in stand-alone studies using the whole seeds [12,24] and seed extracts [13,14,15], but with no effect observed in a study where the *T. foenum-graceum* seed extract was provided as an add-on medication to oral anti-diabetics [20]. Interestingly, reductions in HbA1c were observed in a longer-term study on T2DM [12], indicating that studies of longer duration may be required. This is especially important in a pre-diabetic population as HbA1c may take longer to change due to blood glucose being known to fluctuate between normal and elevated levels [26]. There was also a stabilization of triglyceride levels observed in this study, which has been reported in previous studies of *T. foenum-graceum* seeds in T2DM [11,27]. This indicates that *T. foenum-graceum* may also exert a cardio-protective role in those with glucose dysregulation, although it is unknown if this effect is directly or indirectly due to the insulin sensitizing effects of the *T. foenum-graceum* seed extract.

The effects of *T. foenum-graceum* seeds and seed extracts on glucose metabolism and insulin sensitivity have been attributed to several constituents, including fiber, trigonelline, 4-hydroxyisoleucine (4-HIL), and saponins [28]. It is noteworthy that the extract used in this study was standardized to 20% trigonelline. The use of isolated trigonelline has been shown in an animal model of T2DM to lower blood glucose, free fatty acids, TNF-α and IL-6 levels [29] as well as improve serum insulin, leptin, pancreatic antioxidant status. Additionally, trigonelline was shown to have an insulin-sensitizing effect as measured by homeostasis model of insulin resistance (HOMA-IR) and homeostasis model assessment of β-cell function (HOMA-B). In an in vitro study of HepG2 cells [30], 4-HIL was found to decrease the production of TNF-α and increase the actions of insulin receptor substrate-1 and glucose transporter type 4 (GLUT-4). Previous clinical studies have also found that 4-HIL exerts an insulin secretory and mimicry effect in response to glucose by stimulating pancreatic beta cells [31]. The saponins in *T. foenum-graceum* seeds have been demonstrated in animal studies to decrease FBG and PPBG, insulin and insulin resistance markers [32], IL-6, TNF-α [33], increase expression of GLUT-4 in skeletal muscle [34], and significantly increase pancreatic beta cell function [29]. It is interesting that these constituents of *T. foenum*-*graceum* seeds may support healthy blood glucose levels without inducing hypoglycemia in those with prediabetes, in addition to T2DM, as witnessed in this study.

In addition to effects on glucose, safety measurements for the effect of *T. foenum-graceum* seed extract on those with prediabetes. There were no adverse changes to kidney, liver, and metabolic markers. There was a statistical difference in AST and LD markers between groups at week 12, however this was due to decreased levels in the placebo group rather than an increase in the treatment group. To date, this is the first study to report comprehensive safety data of *T. foenum-graceum* extract use in those with prediabetes. There were no adverse events reported in the long-term *T. foenum-graceum* powdered seed and prediabetes study by Gaddam et al. [17], however no safety measurements were reported. Safety measurements have been reported in those taking *T. foenum-graceum* with T2DM. Whole *T. foenum-graceum* seed studies of 8 weeks duration have reported no adverse changes to kidney and liver markers in those with T2DM [15,19,35]. Benefits including improvements to liver enzyme markers ALT, ALP [19] and AST, as well as systolic blood pressure, blood urea nitrogen levels [15], creatinine and triglycerides [35] were also reported. Safety parameters have been measured in some *T. foenum-graceum* seed extract studies [16,20], with no reported liver or kidney toxicity, however the constituent standardization in these studies was different to the extract used in this study, so results are not fully comparable. Therefore, although there is no indication of any concern about safety, longer studies would be required to assess this extract for longer term use in prediabetics. 

This study was conducted during the COVID-19 pandemic which affected recruitment and participant drop-out rate, resulting in a lower sample size than originally planned. Given the positive results of this small, 3-month study, further investigations with a larger cohort and longer duration are warranted. This study has also highlighted safety issues with the use of a placebo control in those with unmedicated prediabetes over long periods of time.

## 5. Conclusions

The results of this study indicate that this *T. foenum-graceum* seed extract may be an effective treatment for impaired fasting glucose and impaired glucose tolerance, both of which are associated with pre-diabetes. In addition, this *T. foenum-graceum* seed extract may provide cardioprotective support for those with prediabetes by stabilizing triglycerides. 

## Figures and Tables

**Table 1:** Demographics of participants in the Active treatment group and Placebo group at baseline.

	Active Treatment Group			Placebo Group		
	Total	Men	Women	Total	Men	Women
Participant No.	25	16	9	23	12	11
Age (average)	58.2	58.5	54.9	59.7	61.8	65.8
BMI (average)	30.9	31.2	30.4	32.5	33.8	31.2
Previously diagnosed as pre-diabetic (*n*)	14	10	4	8	4	4
Diagnosed as pre-diabetic at screening (*n*)	11	6	5	15	8	7
Taking medication for hypertension (*n*)	8	5	3	5	5	0
Blood pressure -Systolic/Diastolic	129/88	130/89	128/87	128/86	129/88	126/84
Known family history of T2DM (*n*)	15	9	6	16	8	8
Drink alcohol regularly (*n*)	14	11	3	11	6	5
Sedentary lifestyle (*n*)	18	11	7	15	7	8
Exercise regularly (*n*)	8	5	3	8	5	3

**Table 2:** Blood glucose and insulin levels for Active treatment and Placebo groups at baseline, week 6 and week 12.

Test *	Time Point	Group	Mean ± SD	Change from Baseline	*p* Value #	Effect Size	95% CI
Fasting Glucose (<5.5 mmol/L)	Baseline	Active Placebo	6.95 ± 1.80 5.99 ± 0.91	-	0.052	-	-
				-			
	Week 6 Week 12	Active Placebo Active Placebo	6.81 ± 1.74 6.13 ± 0.92 6.52 ± 1.42 6.34 ± 1.12	−0.10 ± 0.72 0.13 ± 0.38 −0.43 ± 0.88 0.35 ± 0.46	0.200 <0.001	0.382 1.108	−0.225–0.984 0.493–1.713
1 h post-prandial glucose (<11.1 mmol/L)	Baseline Week 6 Week 12	Active Placebo Active Placebo Active Placebo	12.32 ± 3.52 11.86 ± 2.90 11.88 ± 3.54 11.90 ± 3.32 11.59 ± 3.42 11.97 ± 3.25	- - −0.37 ± 1.59 −0.03 ± 1.97 −0.74 ± 2.21 0.11 ± 2.30	0.310 0.540 0.200	- 0.193 0.377	- −0.408–0.793 −0.197–0.946
2 h post-prandial glucose (<7.8 mmol/L)	Baseline Week 6 Week 12	Active Placebo Active Placebo Active Placebo	9.15 ± 4.78 8.11 ± 3.97 8.76 ± 4.35 8.92 ± 3.27 8.37 ± 3.83 9.32 ± 3.82	- - −0.18 ± 1.84 0.59 ± 2.13 −0.78 ± 2.74 1.21 ± 2.12	0.210 0.220 0.007	- 0.388 0.806	- −0.220–0.990 0.212–1.392
Fasting Insulin (6–22 mU/L)	Baseline Week 6 Week 12	Active Placebo Active Placebo Active Placebo	18.60 ± 9.69 14.52 ± 6.41 15.80 ± 9.90 12.55 ± 5.94 15.36 ± 7.68 13.74 ± 6.61	- - −2.52 ± 4.50 −1.90 ± 3.22 −3.24 ± 7.38 −0.78 ± 3.42	0.200 0.600 0.140	- 0.157 0.421	- −0.444–0.756 −0.154–0.992
1 h post-prandial insulin (40–90 mU/L)	Baseline Week 6 Week 12	Active Placebo Active Placebo Active Placebo	101.48 ± 55.49 113.00 ± 67.88 112.32 ± 75.45 128.25 ± 127.04 122.32 ± 92.23 117.41 ± 115.31	- - 5.95 ± 50.67 −1.17 ± 58.02 13.26 ± 59.48 −16.00 ± 42.17	0.270 0.690 0.690	- −0.131 −0.563	- −0.761–0.500 −1.164–0.044
2 h post-prandial insulin (15–65 mU/L)	Baseline Week 6 Week 12	Active Placebo Active Placebo Active Placebo	101.76 ± 89.67 76.91 ± 57.67 93.12 ± 97.09 75.10 ± 60.08 91.08 ± 87.91 82.23 ± 49.76	- - −7.43 ± 64.00 −4.42 ± 71.27 −9.54 ± 58.87 3.09 ± 58.71	0.130 0.890 0.470	- 0.215 0.215	- −0.563–0.652 −0.367–0.794

*n* = 25 active group, *n* = 23 placebo group. * Numbers in brackets are healthy reference ranges. # Baseline *p* values are based on difference between group means. Week 6 and Week 12 *p* values are based on difference between group change from baseline.

**Table 3:** HbA1c, C-peptide, lipid profile, atherogenic potential and C-reactive protein for Active treatment and Placebo at baseline and week 12.

Test *	Group	Baseline Mean ± SD	*p* Value	Week 12 Mean ± SD	*p* Value	Effect Size	95% CI
HbA1c % (<6.0%)	Active Placebo	6.03 ± 0.75 5.83 ± 0.58	0.150	5.93 ± 0.78 5.75 ± 0.69	0.410	−0.250	−0.850–0.343
HbA1c mmol/L (<42 mmol/mol)	Active Placebo	42.48 ± 8.13 40.00 ± 6.35	0.130	41.70 ± 8.33 39.60 ± 7.72	0.370	−0.260	−0.861–0.343
C-peptide (0.8–1.9 µg/L)	Active Placebo	0.92 ± 0.36 0.79 ± 0.27	0.075	0.78 ± 0.25 0.81 ± 0.30	0.792	0.082	−0.518–0.681
Cholesterol (3.6–6.9 mmol/L)	Active Placebo	5.31 ± 1.15 5.51 ± 1.29	0.564	5.21 ± 1.02 5.15 ± 1.53	0.875	−0.047	−0.613–0.520
High Density Lipoprotein (HDL) (>0.9 mmol/L)	Active Placebo	1.31 ± 0.31 1.21 ± 0.28	0.241	1.30 ± 0.36 1.18 ± 0.36	0.172	−0.394	−0.964–0.180
Low Density Lipoprotein (LDL) (<2.0 mmol/L)	Active Placebo	3.14 ± 1.17 3.31 ± 1.29	0.643	3.08 ± 1.10 3.11 ± 1.15	0.917	0.030	−0.536–0.597
Total/HDL ratio	Active Placebo	4.28 ± 1.14 4.60 ± 0.90	0.284	4.32 ± 1.13 4.46 ± 0.89	0.633	0.138	−0.430–0.704
Triglycerides (0.3–4.0 mmol/L)	Active Placebo	1.62 ± 0.88 1.97 ± 1.15	0.238	1.58 ± 0.76 2.28 ± 1.28	0.030	0.667	0.081–1.246
C-reactive protein (CRP) (<5 mmol/L)	Active Placebo	5.56 ± 4.14 5.24 ± 4.41	0.797	5.34 ± 4.11 5.00 ± 4.59	0.789	−0.078	−0.644–0.489

*n* = 25 active group, *n* = 23 placebo group. * Numbers in brackets are healthy reference ranges.

**Table 4:** Safety profile and metabolic panel for Active treatment and Placebo at baseline and week 12.

Test *	Baseline			Week 12		
	Active #	Placebo #	*p*-Value	Active #	Placebo #	*p*-Value
Haemoglobin (115–160 g/L)	146 ± 11 (128–165)	145 ± 12 (124–167)	0.807	145 ± 13 (123–166)	141 ± 9 (130–175)	0.225
Red Cell Count (3.6–5.2 × 10^12^/L)	4.9 ± 0.5 (4.4–6.2)	4.9 ± 0.5 (4.2–6.2)	0.717	4.9 ± 0.5 (3.8–5.6)	4.7 ± 0.4 (3.9–5.8)	0.204
Haematocrit (0.33–0.46)	0.45 ± 0.03 (0.39–0.51)	0.44 ± 0.04 (0.38–0.51)	0.645	0.44 ± 0.03 (0.36–0.51)	0.43 ± 0.03 (0.39–0.51)	0.251
Mean Cell Volume (80–98 fL)	91 ± 4.3 (82–100)	91 ± 3.4 (82–97)	0.889	91 ± 4 (86–100)	92 ± 4 (86–102)	0.430
Mean Cell Haemoglobin (27–35 pg)	29.9 ± 1.5 (26–32)	30.1 ± 1.5 (27–33)	0.699	30 ± 2 (28–34)	30.2 ± 1.7 (28–34)	0.515
Platelet Count (150–450 × 10^9^/L)	266 ± 56 (171–385)	260 ± 48 (156–371)	0.697	271 ± 57 (131–326)	253 ± 48 (154–344)	0.264
White Blood Cells (4.0–11.0 × 10^9^/L)	6.6 ± 1.4 (4.4–8.9)	7.2 ± 1.8 (4.1–10.8)	0.230	6.8 ± 1.9 (4.3–9.7)	7.1 ± 1.3 (3.8–12)	0.593
Neutrophils (2.0–7.5 × 10^9^/L)	3.6 ± 0.9 (2.10–5.2)	4.1 ± 1.1 (2.3–8.5)	0.070	3.8 ± 1.7 (2.1–5.8)	4.0 ± 1.0 (1.7–5.6)	0.625
Lymphocytes (1.1–4.0 × 10^9^/L)	2.2 ± 0.6 (1–3.4)	2.3 ± 0.9 (1.1–3.8)	0.674	2.2 ± 0.6 (1.1–3.9)	2.2 ± 0.7 (1.3–5.5)	0.893
Monocytes (0.2–1.0 × 10^9^/L)	0.58 ± 0.14 (0.4–0.8)	0.58 ± 0.24 (0.3–1.4)	0.916	0.66 ± 0.27 (0.4–1.6)	0.59 ± 0.56 (0.3–1.4)	0.363
Eosinophils (0.04–0.40 × 10^9^/L)	0.24 ± 0.17 (0.0–0.7)	0.20 ± 0.17 (0.0–0.7)	0.459	0.24 ± 0.17 (0.07–0.36)	0.18 ± 0.07 (0.0–0.8)	0.143
Basophils (<0.21 × 10^9^/L)	0.06 ± 0.02 (0.01–0.09)	0.11 ± 0.22 (0.0–0.08)	0.248	0.06 ± 0.03 (0.0–0.12)	0.06 ± 0.03 (0.0–0.8)	0.481
Sodium (137–147 × mmol/L)	140 ± 2 (135–145)	139 ± 2 (136–146)	0.253	140 ± 3 (135–146)	140 ± 2 (137–144)	0.643
Potassium (3.5–5.0 × mmol/L)	4.5 ± 0.3 (3.5–5.0)	4.3 ± 0.4 (3.6–5.4)	0.120	4.4 ± 0.3 (3.8–5.0)	4.4 ± 0.3 (4.0–4.8)	0.687
Chloride (96–109 × mmol/L)	105 ± 2 (100–110)	104 ± 2 (99–109)	0.439	105 ± 2 (101–108)	105 ± 3 (101–111)	0.785
Bicarbonate (25–33 × mmol/L)	28 ± 2 (24–32)	28 ± 2 (24–32)	0.489	28 ± 2 (25–31)	27 ± 2 (23–30)	0.420
Creatinine (40–110 × umol/L)	74 ± 11 (51–93)	74 ± 13 (52–93)	0.911	75 ± 12 (47–95)	71 ± 14 (44–95)	0.419
eGFR (>59 mL/min)	81 ± 5 (59–90)	76 ± 7 (65–90)	0.400	81 ± 7 (66–89)	78 ± 8 (63–90)	0.305
Urea (3.0–8.5 mmol/L)	5.92 ± 1.35 (4.5–9.4)	6.18 ± 1.73 (3.5–11.0)	0.560	5.85 ± 1.59 (4.2–9.0)	6.01 ± 1.37 (3.40–9.10)	0.721
Total Bilirubin (2–20 × umol/L)	11 ± 6 (4–28)	10 ± 4 (4–20)	0.415	12 ± 7 (5–36)	10 ± 4 (4–19)	0.125
Alk Phosphatase (ALP) (30–115 × U/L)	73 ± 18 (36–107)	78 ± 18 (43–115)	0.368	71 ± 18 (31–112)	75 ± 19 (37–108)	0.506
Gamma-glutamyl transferase (GGT) (0–45 U/L)	37 ± 23 (11–119)	49 ± 51 (10–262)	0.313	33 ± 16 (10–68)	43 ± 44 (14–208)	0.303
Alanine aminotransferase (ALT) (0–45 U/L)	40 ± 21 (17–110)	35 ± 16 (16–74)	0.437	36 ± 14 (13–68)	29 ± 12 (14–57)	0.083
Aspartate aminotransferase (AST) (0–41 U/L)	30 ± 9 (18–66)	26 ± 7 (15–40)	0.117	30 ± 8 (19–45)	23 ± 7 (12–40)	0.007 ^
Lactate Dehydrogenase (LD) (120–250 U/L)	199 ± 31 (159–269)	183 ± 32 (114–227)	0.081	195 ± 25 (161–254)	170 ± 23 (100–200)	0.001 ^
Calcium (2.25–2.65 × mmol/L)	2.37 ± 0.065 (2.26–2.53)	2.34 ± 0.09 (2.15–2.51)	0.140	2.37 ± 0.07 (2.18–2.48)	2.34 ± 0.07 (2.23–2.49)	0.218
Phosphate (0.8–1.5 × mmol/L)	1.14 ± 0.16 (0.7–1.4)	1.09 ± 0.20 (0.8–1.5)	0.358	1.11 ± 0.14 (0.8–1.3)	1.17 ± 0.22 (0.7–1.5)	0.252
Total Protein (60–82 × g/L)	71 ± 4 (64–79)	70 ± 4 (66–78)	0.632	70 ± 3 (62–76)	69 ± 3 (63–76)	0.128
Albumin (35–50 × g/L)	42 ± 2 (39–47)	42 ± 2 (38–47)	0.640	42 ± 2 (38–47)	42 ± 2 (37–45)	0.439
Globulin (20–40 × g/L)	28 ± 5 (21–36)	28 ± 3 (23–32)	0.634	28 ± 3 (20–34)	26 ± 5 (5–31)	0.144

*n* = 25 active group, *n* = 23 placebo group. * Numbers in brackets are healthy reference ranges. # Data written as mean ± SD with range (in brackets). ^ AST and LD levels, whilst significantly different, were all within Healthy Reference Range, so unlikely to be clinically significant.

## Data Availability

Date described in the manuscript will be made available pending application and approval.
